# Web-based bioinformatics workflows for end-to-end RNA-seq data computation and analysis in agricultural animal species

**DOI:** 10.1186/s12864-016-3118-z

**Published:** 2016-09-27

**Authors:** Weizhong Li, R. Alexander Richter, Yunsup Jung, Qiyun Zhu, Robert W. Li

**Affiliations:** 1 J. Craig Venter Institute, La Jolla, CA 92037 USA; 2 United States Department of Agriculture, Agriculture Research Service (USDA-ARS), Animal Genomics and Improvement Laboratory, Beltsville, MD 20705 USA

**Keywords:** RNA-seq, Animal genomes, Workflow, Mapping, Assembly, Transcript quantification

## Abstract

**Background:**

Remarkable advances in Next Generation Sequencing (NGS) technologies, bioinformatics algorithms and computational technologies have significantly accelerated genomic research. However, complicated NGS data analysis still remains as a major bottleneck. RNA-seq, as one of the major area in the NGS field, also confronts great challenges in data analysis.

**Results:**

To address the challenges in RNA-seq data analysis, we developed a web portal that offers three integrated workflows that can perform end-to-end compute and analysis, including sequence quality control, read-mapping, transcriptome assembly, reconstruction and quantification, and differential analysis. The first workflow utilizes Tuxedo (Tophat, Cufflink, Cuffmerge and Cuffdiff suite of tools). The second workflow deploys Trinity for *de novo* assembly and uses RSEM for transcript quantification and EdgeR for differential analysis. The third combines STAR, RSEM, and EdgeR for data analysis. All these workflows support multiple samples and multiple groups of samples and perform differential analysis between groups in a single workflow job submission. The calculated results are available for download and post-analysis. The supported animal species include chicken, cow, duck, goat, pig, horse, rabbit, sheep, turkey, as well as several other model organisms including yeast, *C. elegans*, *Drosophila*, and human, with genomic sequences and annotations obtained from ENSEMBL.

The RNA-seq portal is freely available from http://weizhongli-lab.org/RNA-seq.

**Conclusions:**

The web portal offers not only bioinformatics software, workflows, computation and reference data, but also an integrated environment for complex RNA-seq data analysis for agricultural animal species. In this project, our aim is not to develop new RNA-seq tools, but to build web workflows for using popular existing RNA-seq methods and make these tools more accessible to the communities.

## Background

Remarkable advances in Next Generation Sequencing (NGS) technologies [1] and computational theory and practice as well as rapid developments of bioinformatics algorithms in recent years have significantly accelerated genomic researches.

Sequencing steady-state RNA in a biological sample (RNA-seq) [2, 3], as one of the major NGS approaches, has been widely used in many fields. RNA-seq overcomes many limitations of previous technologies, such as microarrays and real-time PCR. Most importantly, RNA-seq has been shown to unravel previously inaccessible complexities in the transcriptome, such as allele-specific expression and novel promoters and isoforms, gene expression (abundance estimation), detection of alternative splicing, RNA editing, and novel transcripts.

In the past years, many tools and methods have been developed for RNA-seq data analysis. Some major categories of these tools including read-mapping, transcriptome assembly or reconstruction, and expression quantification [4].

Aligning RNA-seq reads against a reference genome or transcriptome (a.k.a read-mapping) is the most common job when a reference is available. There are a large number of general purpose aligners available such as Bowtie [5, 6], BWA [7, 8], SOAP [9, 10], ZOOM [11], SHRiMP [12] and many others. Programs such as TopHat [13], GSNAP [14], MapSplice [15], QPALMA [16], STAR [17] and HISAT [18] are RNA-seq specific aligners, which are capable of identifying splicing events.

Transcriptome reconstruction or RNA-seq assembly is another route to analyze RNA-seq data. This can be performed with or without a reference genome. Scripture [19] and Cufflinks [20] are examples of reference genome dependent programs. They take mapping alignments to a reference genome as the input. Oasis [21], TransABySS [22] and Trinity [23] are *de novo* assemblers that don’t require reference genomes.

Mapping and assembly are relatively computation-intensive jobs, which supply data for downstream expression quantification using programs such as Cufflinks [20], MISO [24] and RSEM [25]. For multiple RNA-seq datasets under different conditions, differential expression can be analyzed with Cuffdiff [20], DegSeq [26], EdgeR [27], DESeq [28] and several other methods.

To make sense of RNA-seq data, a full analysis pipeline usually requires multiple procedures and different tools. Besides the RNA-seq specific tools discussed above, many other NGS data processing tools are also required such as SolexQA [29] and Trimmomatic [30] for sequence quality control, Samtools [31] and Bedtools [32] for alignment file processing.

Difficulties in creating these complicated computational pipelines, installing and maintaining software packages, and obtaining sufficient computational resources all tend to overwhelm bench biologists from attempting to analyze their own RNA-seq data. So, despite the availability of the great set of computational tools and methods for RNA-seq data analysis, it is still very challenging for a biologist to deploy these tools, integrate them into workable pipelines, find accessible computational platforms, configure the compute environment, and perform the actual analysis.

Today, RNA-seq has been widely used in animal studies, so developing integrated bioinformatics systems specific to agricultural species, especially easy-to-use web portals, is of great importance for researchers in the agricultural community.

To this end, we have developed a web portal offering integrated workflows that can perform end-to-end compute and analysis, including sequence (Quality Control) QC, read-mapping, transcriptome assembly, reconstruction and quantification, and multiple analysis tools. The first workflow utilizes the Tuxedo suite of tools (Tophat, Cufflink, Cuffmerge and Cuffdiff) [33] for comparative reference-based analysis. The second workflow deploys Trinity [34] for *de novo* assembly, RSEM [25] for transcript quantification, and EdgeR [27] for differential analysis. The third combines STAR [17], RSEM and EdgeR for data analysis. All these workflows support multiple samples and multiple groups of samples and perform differential analysis between groups in a single workflow job submission. The RNA-seq portal is freely available from http://weizhongli-lab.org/RNA-seq for all users. The backend software package is also available as open source software.

## Implementation

The portal is implemented with several state-of-the-art High Performance Computing (HPC), workflow and web development software tools including Galaxy [35], StarCluster (http://star.mit.edu/cluster/docs/latest/index.html), running on modern scalable cloud compute and storage sources from Amazon Web Services (AWS).

**Fig. 1:**
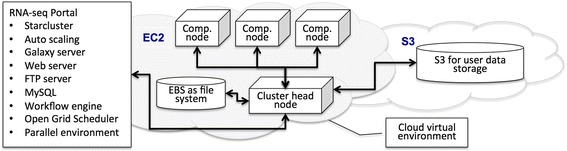
Cyber framework of the RNA-seq portal

The system is illustrated in Fig. 1. The whole computer system supporting the RNA-seq portal resides in the AWS cloud environment. A virtual computer cluster consists of a head node and compute nodes is controlled by StarCluster software. The initial one-time launch of the virtual computer cluster is performed from a desktop or laptop where StarCluster software is installed and configured with our StarCluster configuration file. The virtual computer cluster’s head node is running all the time. It serves as the portal’s front end and provides web server, FTP server and Galaxy server for users to interact with the portal. Compute nodes are automatically brought online or shutdown according to the need of user jobs. An EBS volume, which provides fast access and persistent data storage, is used as a shared file system for the virtual computer cluster. S3 storage, which provides cost-effective data storage, is used to store computed user data.

### Cluster head node

Once the head node is up and running, the virtual cluster can be controlled within this head node, where StarCluster software is also installed. The virtual cluster is configured with Open Grid Engine (OGE) job scheduling system with parallel environment enabled. All user-submitted jobs will be managed by the OGE. The StarCluster auto-scaling script, which runs in the background on the head node, automatically starts up new compute nodes when jobs are waiting in the OGE queue and shuts them down when the queue empties, reducing compute costs.

An Apache web server runs on the head node. It supports the RNA-seq portal website and provides reference genome data and user data download. An FTP server also runs on the head node, allowing users to download reference genome data and upload user data. A MySQL server is used in tracking user jobs and supporting the Galaxy server. The RNA-seq portal documentation is supported by a DokuWiki server.

### Galaxy server

Galaxy [35] is a web-based platform that supports data intensive biomedical research through Galaxy enabled tools and workflows. In recent years, Galaxy has been widely used by the community. The main Galaxy project server along with many other public galaxy servers offers many computational tools for users to perform data analysis and provides friendly environment and interface for users to manage jobs and data using web browsers. In this project, we run a Galaxy server instance for user management and as a portal where users can upload data and run the workflows we implement.

### Workflow engine

RNA-seq data analysis requires workflows with multiple procedures and many different tools. The tools all have different requirement in computer memory, I/O speed, disk space, network bandwidth, density of computing cores, parallel environment settings etc. So given a computer grid or cloud infrastructure, it is not trivial to make a fully automated workflow that meets the requirements of all distinct tools and maximize the usage of provided compute resources.

The Galaxy platform supports running individual compute tools and also supports workflow integration. However, the workflow function offered by Galaxy requires users to have relatively deep knowledge of Galaxy software and the tools being integrated into the workflows, so it is quite difficult for common users to fully take advantage of the Galaxy workflow capacity.

In this project, we provided users with pre-configured workflows, which are launched as standalone tools from the Galaxy interface. The workflows in this project are configured with a lightweight workflow engine we developed in our earlier projects [36], supported by the Human Microbiome Project (HMP).

## Results and Discussion

The RNA-seq portal offers three integrated workflows. All these workflows are implemented so that a user can run multiple groups of samples under different conditions (e.g. case and control, or time series) with a single job submission. A workflow will perform identical process (e.g. read-mapping) for each individual sample, then compare results between groups, and can also analyze data based on pooled samples or groups.

### Tuxedo (Tophat, Cufflink, Cuffmerge and Cuffdiff) workflow

**Fig. 2:**
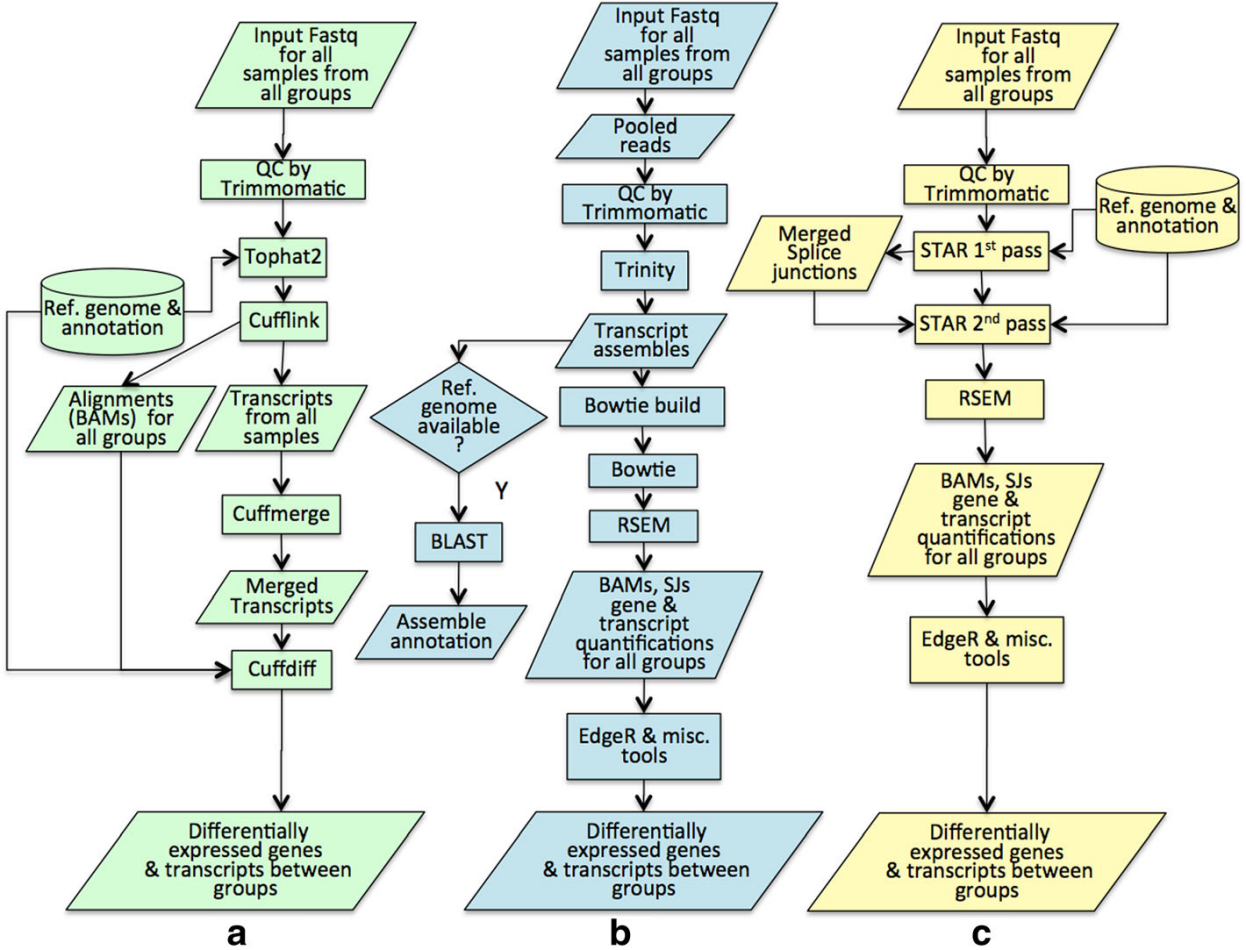
Flowchart of the three workflows: **a** Tuxedo workflow, **b** Trinity de-novo assembly and post-analysis workflow, and **c** STAR mapping and post analysis workflow

The Tophat, Cufflink, Cuffmerge and Cuffdiff workflow, also know as the Tuxedo Package [33], is one of the most widely used tools in RNA-seq data analysis. The workflow we implemented here is largely based on the pipeline described in the Tuxedo publication [33]. The pipeline is shown in Fig. 2a.

Given user input sequence files in FASTQ format for several groups of samples, the workflow first runs a three step sub-workflow for each individual: (1) Sequence QC: for either Paired End (PE) or Single End (SE) read input, remove low quality reads and trim low quality bases using Trimmomatic [30] with default parameters; (2) Reference-based alignment: align cleaned reads to a selected reference genome with Tophat; and (3) Transcript Assembly: assemble the transcripts with Cufflink.

The results of this process are then combined into a single merged transcriptome annotation with Cuffmerge. Finally, for each pair of sample groups, Cuffdiff is used to identify differentially expressed genes and transcripts between them.

### Trinity de-novo assembly and post-analysis workflow

This workflow is implemented according to the Trinity protocol [34]. Additional information about the protocol is described at http://trinityrnaseq.github.io. The structure is outlined in Fig. 2b.

This workflow first uses Trinity to assemble all samples together into a combined transcriptome. It then indexes the transcriptome sequences using bowtie and annotates transcripts by comparing them to cDNA sequences from reference genomes using BLASTN [37]. Trinity itself has a QC component, so we rely on Trinity’s own QC procedure for sequence cleaning. After transcript assembly, the workflow aligns high-quality reads from each sample back to the assembled transcript using Bowtie, then performs transcript quantification using RSEM [25]. Finally, the workflow runs pair-wise differential analysis with EdgeR [27] using the scripts available from Trinity package.

### STAR mapping and post analysis workflow

This workflow uses STAR [17], an ultrafast RNA-seq aligner for mapping reads to a reference genome (Fig. 2c). Similar to the Tuxedo workflow, the STAR workflow first performs sequence QC using Trimmomatic, runs STAR’s first pass mapping to a reference genome for each sample. Splice junctions identified there are then pooled and used to map the high quality reads from each sample one more time with STAR’s second pass mapping to produce a new set of alignments, splice junctions and other results. These are then used to generate gene and transcript quantification results with RSEM. Finally, the workflow runs pair-wise differential analysis with EdgeR. Here, we use a set of scripts provided in Trinity package to perform EdgeR and to call several other functions.

### Choice of workflows

Tuxedo and STAR workflows are reference genome/transcriptome based approaches. When reference genomes are available and the main goal is to quantify the expression level of known genes and transcripts, then these two workflows are the choice. Tophat2 and STAR are both very popular aligners. Regarding the accuracy and performance, they have been extensively evaluated, compared and discussed along with many other aligners in algorithm papers and in reviews [17, 18, 38, 39], as well as in public forums (e.g. seqanswers.com). Between Tophat2 and STAR, none is significantly better than the other in all aspects (e.g. number of mapped reads, junctions, false calls, etc), except that STAR is much faster than Tophat2 and Tophat2 uses much less Memory. Given the current availability of high RAM computers, the overall compute cost of STAR is significantly lower than Tophat2. It is importantly to understand their pros and cons by check these paper and resources in using the two workflows and interpret their results.

When there is no reference genome available or the reference genome is poorly assembled or annotated, Trinity workflow can be utilized in RNA-seq analysis. This is important for many non-model organisms or cancer samples.

Given the convenience of our web portal job submission, it is possible for users to run multiple workflows on the same dataset once the input data are uploaded to users’ workplace. That way, it is possible to compare the results to see whether consistent observations can be obtained with different approaches, to identify questionable results, and to look for method specific predictions.

### Portal interface

**Fig. 3:**
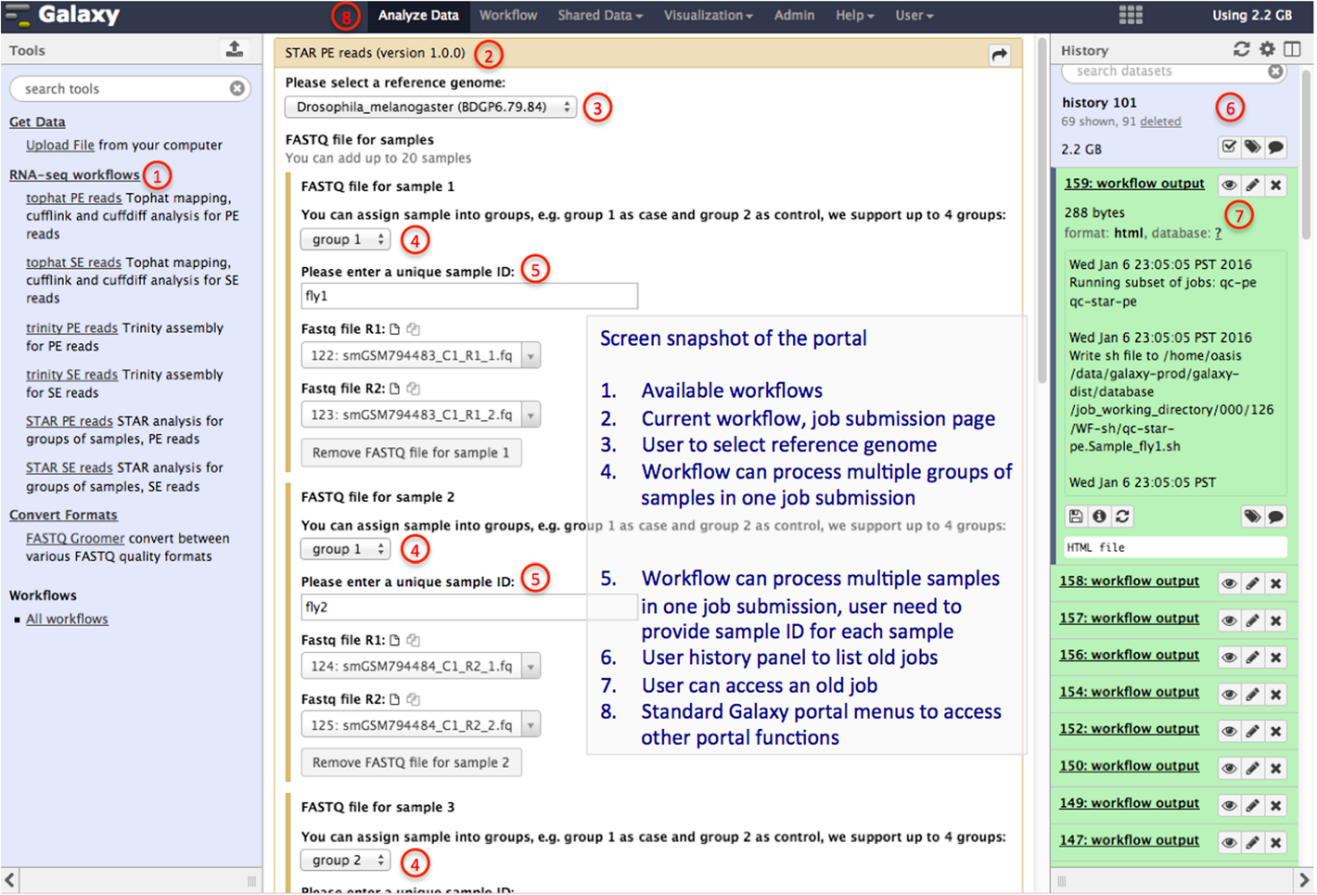
Screenshot of the RNA-seq portal job submission page

The web portal to run the workflows (see Fig. 3 for a screenshot) is implemented with Galaxy framework. We did only necessary customization to the Galaxy page so that the layout of the portal page is very similar to the official public Galaxy server and therefore users with prior experiences with Galaxy can easily start to use our resources. Users new to Galaxy are recommended to learn Galaxy’s concept and know the basic usage before submitting jobs to the portal.

The workflow saves all major output files from each step of the workflow so that users can access not only the final results but also all intermediate data. For example, all alignment outputs in BAM format are saved and these BAM files have been sorted by the workflow to assist users’ later analysis. When the workflow is completed, users can download a gzipped file that contains all the results from their analysis or browse and access each individual file from the RNA-seq portal.

Some results can be directly used from our server. For example, users can directly load data (e.g. BAM alignments) into an instance of the Integrative Genomics Viewer (IGV) [40] by providing the web URL of the file from our server. We have pre-loaded genomes and annotations for all the species in our portal to support public IGV instances so it is easy to visualize and explore data from our pipeline. More detailed documention is available from the RNA-seq portal.

### Reference genomes

The workflows support important animal species including chicken, cow, duck, goat, pig, horse, rabbit, sheep, turkey, as wells as human, mouse and several other model organisms: yeast, *C. elegans*, *Drosophila*, and others (Table 1). ENSEMBL [41] is used as the primary source for genome data, except for goat which was obtained from the International Goat Genome Consortium (IGGC) [42]. We downloaded the genome, gene, and peptide sequences, as well as gene models (GTF files) for each genome. These were formatted and indexed with all of bwa, bowtie2, STAR, RESM, BLASTN, BLASTP and IGV, for use in all the workflows from the portal. All are available for download through both our web and FTP servers if users want to perform down-stream analysis on their own systems. Current genomics resources are based on Ensembl release 84. We plan to update the databases every 6 months. With each update, the new databases will replace the last set of databases in all workflows. But we will make last set of databases available for user download.

**Table 1:** A list of genomes supported by the workflows

Name	Species	Ensembl/IGGC build
Chicken	*Gallus gallus*	Galgal4.84
Duck	*Anas platyrhynchos*	BGI_duck_1.0.84
Cow	*Bos taurus*	UMD3.1.84
Goat	*Capra hircus*	goat_scaffoldFG_V1.1
Pig	*Sus scrofa*	Sscrofa10.2.84
Horse	*Equus caballus*	EquCab2.84
Rabbit	*Oryctolagus cuniculus*	OryCun2.0.84
Sheep	*Ovis aries*	Oar_v3.1.84
Turkey	*Meleagris gallopavo*	UMD2.84
Yeast	*Saccharomyces cerevisiae*	R64-1-1.84
Nematode	*Caenorhabditis elegans*	WBcel235.84
Fruitfly	*Drosophila*_*melanogaster*	BDGP6.84
Mouse	*mus*_*musculus*	GRCm38.84
Human	*Homo sapiens*	GRCh38.84

## Conclusions

In order to assist researchers in the RNA-seq field to deal with data analysis challenges, we implemented the RNA-seq web portal with three integrated workflows, which can be used for end-to-end RNA-seq data compute and analysis. RNA-seq is a very active field with many great analysis tools. Our web portal makes available tools more accessible to the broader research community using RNA-seq technology but without access to either compute resources or expertise in bioinformatics. The tools, such as Tuxedo, Trinity and STAR, are all well-tested and established tools set up with standard analysis protocols. This is especially beneficial for researchers who are new to RNA-seq data analysis. We plan to add additional tools and workflows based on users’ need or the available new tools (e.g. HISAT [18]).

To support users who prefer to run these workflows locally or want to setup web portal on their own servers, with the flexibility of using different parameters, our backend software package is available as open source software. The software package needs to be installed on generic Linux computer clusters that support Open Grid Engine. These systems are widely available from HPC facilities in Universities and institutions, as well as from Cloud providers (e.g. Amazon Web Services). The installation documents are available from our project page at http://weizhongli-lab.org/RNA-seq.

## Availability and requirements

- Project name: RNA-seq web portal for animal species
- Project home page: http://weizhongli-lab.org/RNA-seq.
- Operating system(s): Platform independent
- Programming language: Perl (client-side scripts)
- Other requirements: web browsers
- License: no license needed
- Any restrictions to use by non-academics: no restriction

## Abbreviations

AWS: Amazon Web Services
HPC: High Performance Computing
IGGC: International Goat Genome Consortium
NGS: Next generation sequencing
OGE: Open Grid Engine
QC: Quality control
